# Supplementary material for: Toxoplasma gondii Serotypes in Italian and Foreign Populations: A Cross-Sectional Study Using a Homemade ELISA Test
Source: Microorganisms. 2022 Aug 5;10(8):1577. doi: 10.3390/microorganisms10081577 (PMC9415598; doi:10.3390/microorganisms10081577)
Supplement: Supplementary file 1 [file microorganisms-10-01577-s001.zip › microorganisms-1804507-supplementary.pdf]

Supplementary materials

Supplementary Table S1. The geographic origins of the population studied.

	Region									
Country of Birth	EU		Asia		South America		Africa		Total	
	n	%	n	%	n	%	n	%	n	%
ALBANIA	1	0.5%	0		0		0		1	0.5%
BANGLADESH	0		2	1.1%	0		0		2	1.1%
BOLIVIA	0		0		1	0.5%	0		1	0.5%
BRAZIL	0		0		9	4.8%	0		9	4.8%
CAMEROON	0		0		0		3	1.6%	3	1.6%
CENTRAL AFRICAN REPUBLIC	0		0		0		1	0.5%	1	0.5%
COLOMBIA	0		0		5	2.7%	0		5	2.7%
DEMOCRATIC REPUBLIC OF CONGO	0		0		0		2	1.1%	2	1.1%
IVORY COAST	0		0		0		3	1.6%	3	1.6%
CUBA	0		0		1	0.5%	0		1	0.5%
ECUADOR	0		0		1	0.5%	0		1	0.5%
GAMBIA	0		0		0		2	1.1%	2	1.1%
GHANA	0		0		0		11	5.9%	11	5.9%
GREAT BRITAIN	1	0.5%	0		0		0		1	0.5%
GUINEA	0		0		0		3	1.6%	3	1.6%
GUINEA BISSAU	0		0		0		5	2.7%	5	2.7%
EQUATORIAL GUINEA	0		0		0		1	0.5%	1	0.5%
ITALY	103	54.8%	0		0		0		103	54.8%
LEBANON	0		1	0.5%	0		0		1	0.5%
MALI	0		0		0		2	1.1%	2	1.1%
MOROCCO	0		0		0		3	1.6%	3	1.6%
MOLDAVIA	1	0.5%	0		0		0		1	0.5%
NEPAL	0		1	0.5%	0		0		1	0.5%
NIGER	0		0		0		1	0.5%	1	0.5%
NIGERIA	0		0		0		8	4.3%	8	4.3%
NIGERIA (FED.)	0		0		0		1	0.5%	1	0.5%
PARAGUAY	0		0		1	0.5%	0		1	0.5%
PORTUGAL	1	0.5%	0		0		0		1	0.5%
DOMINICAN REPUBLIC	0		0		1	0.5%	0		1	0.5%
ROMANIA	5	2.7%	0		0		0		5	2.7%
RUSSIA	0		1	0.5%	0		0		1	0.5%
SENEGAL	0		0		0		2	1.1%	2	1.1%
SIERRA LEONE	0		0		0		1	0.5%	1	0.5%
SWITZERLAND	1	0.5%	0		0		0		1	0.5%
TUNISIA	0		0		0		1	0.5%	1	0.5%
Total	113	60.1%	5	2.7%	20	10.6%	50	26.6%	188	100.0%
